# MAIT Cells in Barrier Tissues: Lessons from Immediate Neighbors

**DOI:** 10.3389/fimmu.2020.584521

**Published:** 2020-11-30

**Authors:** Ali Amini, Declan Pang, Carl-Philipp Hackstein, Paul Klenerman

**Affiliations:** ^1^ Translational Gastroenterology Unit, Nuffield Department of Medicine, University of Oxford, Oxford, United Kingdom; ^2^ Peter Medawar Building for Pathogen Research, University of Oxford, Oxford, United Kingdom

**Keywords:** mucosal-associated invariant T cells, microenvironment, microbiome, metabolism, tissue resident cells, mucosal immunology, diet

## Abstract

Mucosal-associated invariant T (MAIT) cells are innate-like T cells present at considerable frequencies in human blood and barrier tissues, armed with an expanding array of effector functions in response to homeostatic perturbations. Analogous to other barrier immune cells, their phenotype and function is driven by crosstalk with host and dynamic environmental factors, most pertinently the microbiome. Given their distribution, they must function in diverse extracellular milieus. Tissue-specific and adapted functions of barrier immune cells are shaped by transcriptional programs and regulated through a blend of local cellular, inflammatory, physiological, and metabolic mediators unique to each microenvironment. This review compares the phenotype and function of MAIT cells with other barrier immune cells, highlighting potential areas for future exploration. Appreciation of MAIT cell biology within tissues is crucial to understanding their niche in health and disease.

## Introduction

Mucosal-associated invariant T (MAIT) cells have been intertwined with barrier immunity ever since they were coined (1, 2). Abundant in human blood, making up to 10% of peripheral T cells, they are also enriched in tissues (2–4). MAIT cells are defined as *TRAV1-2^+^* (TCR Vα7.2^+^) T cells restricted by the MHC class I-related molecule (MR1), which recognizes non-peptide riboflavin biosynthesis intermediates conserved among bacteria and fungi (5–10). All mammalian barrier surfaces are colonized by riboflavin-synthesizing commensals, and MR1 has co-evolved with MAIT cells through most mammalian evolution (11), thus being an example of barrier surfaces imprinting human immunity.

Barrier surfaces are sites of cross-talk between the host and diverse external environments. MAIT cells are found in the intestine, skin, respiratory, oral and female genital mucosa, which all house microbial communities adapted to the local environment and in symbiosis with the host (12) (**Table 1**). MAIT cells are dependent on this microbiome and are part of a community of tissue immune cells anatomically close to epithelial surfaces (20, 35), all poised for rapid effector functions to maintain tissue homeostasis (36, 37).

**Table 1:** MAIT cells in healthy human barrier tissues.

Barrier tissue MAIT location	Frequency	Phenotype and activation	Ref
**Oral**			
*Buccal mucosa* Close to basement membrane, both within the epithelial layer and connective tissue.	No enrichment compared to blood (50% of oral CD8αα T cells)	↑CD103^+^ (20-80%) – most are CD8^+^ ↑HLA-DR, CD69, PD-1, CTLA-4 ↓CD38, perforin PMA/ionomycin stim: ↑IL-17A, GzmB (esp. CD103^+^MAIT) ↓TNF, IFN-γ	(13, 14)
**Gut**			
*Gastric mucosa* Lamina propria	LPMC 2% (0-12%) Gastric 1% (0.4-2.39)	↑CD103^+^CD69^+^ (80%)	(15) *(16)
*Duodenum* Lamina propria and epithelium	1.7% IEL 3% (0.1-5%) LPL 2% (0.5-4%)	↓IL-18Rα IL-12+IL-18 stim: ↓IFN-γ	(17, 18)
*Jejunum* Lamina propria and epithelium	IEL *60% (n = 1*): *(*Vα7.2^+^ T cells)		(6)
*Colon* Lamina propria and epithelium	No data in healthy Cancer: IEL 1%; LPL 2% Inactive UC 12% (30% of CD8^+^ are MDR1+)	Most CD8^+^ CD103^+^ *(n = 1)* ↑CD69 (>90%), HLA-DR, CD25, TIGIT, PD-1, CTLA-4, LAG-3 ↑GzmB (baseline) ↑Tbet, RORγt PMA/ionomycin stim: ↑TNF, IFN-γ, IL-17A, IL-22 E coli stim: ↓IFN-γ	*(19) (20–22)
*Rectum*	2% (0.5-8%)	CD8^+^ > DN MAIT ↑IL-23R, CSF1, TNF, CD40LG, CRTAM ↓GzmK	(23, 24)
*Small intestine* *Fetal 2^nd^ trimester*	0.5% (0.2-1%)	CD8α (30%), Ki67 (20%) PMA/ionomycin stim: ↑IL-22 E coli stim: ↑IL-22, ↓IFN-γ	(25)
**Lung**			
*Bronchial tree* Epithelium > lamina propria	5% (Endobronchial biopsy) *TRAV1-2^+^%CD8*: Trachea (42%*)* > proximal (35%*):* > distal bronchus (22%) (*n = 1)*		(26) (27)
*Lung parenchyma*	6% [TRAV1-2^+^%CD8]		(27)
*Lung parenchyma Fetal 2^nd^ trimester*	0.8% (range 0.6-2)	CD127^+^IL-18Rα^+^ (>90%) CD8α (20%), Ki67 (15%) E coli stim: ↑IL-22, ↓IFN-γ	(25)
*Sputum*	2%		(26)
*BAL adult*	2% *4% [TRAV1-2^+^%CD8]*	↑CD103^+^ (75%), PMA/ionomycin stim: ↑IL-17A (esp. CD103^+^)	(26, 28) (27)
*BAL children*	1% 3% (in CAP)	Most CD103^−^ – in CAP >50% CD103^+^ Plasma: ↑IL-12p70 ↓IFN-γ, IL-22, IL-23, MIP-1α, MIP-1β CAP: ↑IL-17A, IL-22, IL-23, IL-1β, IL-6, IL-12p70, MCP-1, MIP-1α, MIP-1β ↑ IL-17A: IFN-γ ratio ↑ *HIF1A, AHR, BATF, PLZF* ↓*TCF7*	*(29)
**Skin**			
*Epidermis and dermis*, especially papillary dermis and adjacent to the superior vascular plexus	3.8+/-0.32% (by IF) Epidermis: 1.5±0.5% (11.6±11.0% of CD8^+^) Dermis: 0.5±0.1% (4.6±4.0% of CD8^+^)	↑CLA^+^ (80%) ↑CD103^+^(80% epidermis, 40% dermis)	(30) *(31)
**Female Genital Tract**			
*Endometrium* Lamina propria close to and within glandular epithelium	1% (range 0-3%)	↓PLZF (DN vs CD8^+^ MAIT ↓ Tbet; ↑PLZF, RORγt, Helios) E coli stim: ↑polyfunctional, IL17-A, IL-22 ↓IFN-γ, TNF, GzmB	(32, 33)
*Cervix* Endocervix, adjacent to simple columnar epithelium; Transformation zone lamina propria; Ectocervix on both side of basement membrane, predominantly in clusters within epithelium	2% (range 0-6%)	↓Eomes	(32)
*Placenta* Decidua parietalis	2% (IVB 4%)	↑CD69 (80%), CD25 (25%), HLA-DR (35%), PD-1 (70%), Ki67 (15%) ↓CD127 (50%) E coli stim: ↑GzmB, Perforin	(34)

Location, frequency, phenotype, and function of MAIT cells in healthy human tissue compiled from studies to date. Frequency is expressed as a % of total CD3^+^ unless otherwise specified. Studies highlighted (*) defined MAIT cells using MR1-tetramer. All other studies used proxy measures of variable stringency to identify MAIT cells, predominantly CD161^+^Va7.2^+^. Enrichment and comparisons of phenotype or function are compared to blood. BAL, bronchoalveolar lavage; Ca, cancer; CAP, community acquired pneumonia; DN, double negative; GzmB, granzyme B; DP, decidua parietalis; DN, double negative; IEL, intraepithelial lymphocytes; IF, immunofluorescence; IVB, intervillous blood; LPL, lamina propria lymphocytes.

**Figure 1:**
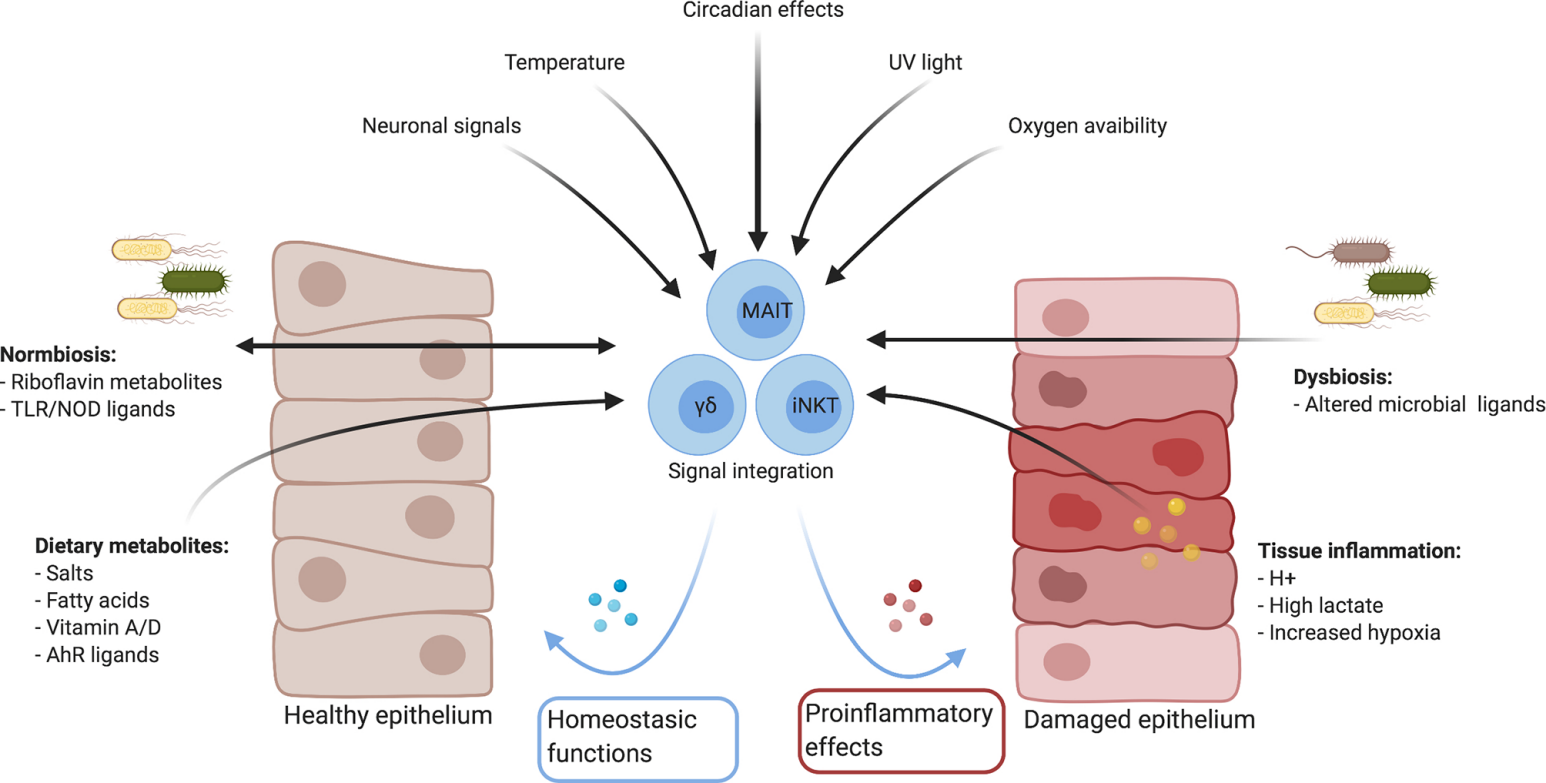
Summary of mucosal environmental factors which could influence immune responses directly and indirectly. Created with Biorender.com.

The range of MAIT cell effector functions is only just being explored. It is increasingly appreciated that resident macrophages and lymphocytes are in constant cross-talk with tissues, integrating cues from the local microbiome, cellular, environmental and metabolic milieus for their development and function (38–44). In this review, we show that MAIT cells occupy a similar niche, engage in similar cross-talk and could sense similar factors (**Figure 1**, **Table 2**). Furthermore, we draw parallels with other barrier lymphocytes to explore tissue-specific factors which could modulate their function at mucosal surfaces, with the hypothesis that there are unexplored functional and metabolic adaptions for their survival and execution of tissue-specific effector functions. Understanding these factors is important to expand our knowledge of their role in shaping tissue function in health and disease.

**Table 2:** Environmental factors and sensors.

		Protein Atlas	Fergusson (45)	Park (46)	Salou (47)	Hinks (48)	Hinks TCR (48)	Lamichhane E.coli (49)	Leng TCR (20)	Sharma anti-CD3 (50)	Sharma BCG (50)	Lu CAP (29)
**Physical factors**	Sensor*											
Hypoxia	HIF1A (effector)						↑	↑		↑	↑	BAL > Blood MAIT17
Acidosis	GPR65		↑	↑	↑	↑				↑	↑	MAIT17
Osmolarity	NFAT5						↑		↑	↑	↑	
Mechanical	PIEZO1					↑						
**Metabolite**												
Vitamin A	RARG	↑		↑	↑	↑				↑		
Vitamin D	VDR						↑	↑	↑		↑	
Lactate	SLC16A1(*transporter*)						↑	↑	↑			
Tryptophan metabolites	AHR									↑		BAL > Blood
	GPR35				↑	↑						
Oxysterols	GPR183							↑				MAIT1
Purines	P2RX7									↓		
	P2RY14				↑	↑			↑			
**Neuropeptide**												
Noradrenaline	ADRB2		↑	↑	↑	↑	↓		↓			
Neuromedin U	NMUR1	↑			↑		↓					
BigLEN	GPR171	↑			↑	↑	↑	↑		↑	↑	

Examples of tissue factors which could modify human MAIT cells in an analogous manner to other resident immune cells. Transcriptional expression of purported sensors*, in some cases including relevant non-sensor genes. All transcriptional datasets are from human blood MAIT cells, except (29) which is from matched BAL and blood MAIT cells. Protein atlas, genes enriched in MAIT cells compared to other blood immune cells; Fergusson (45), genes enriched in CD161^+^ T cells; Park (46), genes enriched in CD161^+^ Va7.2^+^ compared to conventional T cells; Salou (47), genes enriched in MAIT cells compared to conventional CD8^+^ T cells; Hinks (48), genes enriched in MR1-tetramer^+^ MAIT cells compared to CD8^+^ T cells; Hinks TCR (48), genes upregulated on 5-OP-RU activation of MAIT cells; Lamichhane E coli (49), genes upregulated on E coli activation of MAIT cells; Leng (20), genes enriched in MAIT cells stimulated with TCR-dynabeads; Sharma (50), genes upregulated with anti-CD3 or BCG stimulation; Lu (29), genes enriched in BAL MAIT cells from children with community acquired pneumonia (CAP). MAIT17 = type-17 MAIT cells; MAIT1 = type-1 MAIT cells.

## Barrier Tissues

Mammalian barrier surfaces are diverse physiological, chemical and cellular niches. The skin is dry, lipid-rich and acidic, primarily due to epidermal fatty acids, with unique exposures to high-salt content, UV-radiation and large fluctuations in temperature (51, 52). The female genital tract is even more stressed, driven by *Lactobacillus sp.*-derived lactic acid and hydrogen peroxide, with additional structural and immunological changes with the menstrual cycle, pregnancy and age (12, 53). The digestive system varies along its length, both in terms of physical factors and the unique composition of the microbiome: the oral mucosa is subject to occasional physical trauma from mastication, while the gut has the largest microbial biomass in the body in addition to a nutrient rich environment consisting of bile acids and diet-derived metabolites. Finally, the lungs are subject to fluctuating mechanical shear stress from ventilation and gravitational posture dependent changes, and also differ in structure and microbial sterility from higher to lower order bronchi and alveoli (54).

Most sites have underlying dense neural networks and varying concentrations of physiological parameters such as oxygen tension, lactate, glucose and amino acids depending on the specific location and inflammatory context; relative hypoxia is physiological in healthy skin hair follicles and the intestinal lumen, and induced in the face of increasing tissue demand during inflammation in all tissues. The inflammatory tissue microenvironment is further dysregulated by the catabolic processes necessary for generating an immune response, through excess nutrient consumption and generation of potentially toxic metabolic by-products. Moreover, these physical and biochemical changes interact with and shape the microbiome, therefore directly and indirectly interacting with resident immune cells, including MAIT cells.

## Barrier MAIT Cells

Barrier tissue lymphocytes include innate, innate-like and adaptive immune cells (37). Activation is antigen-independent in natural killer (NK) and innate lymphoid cells (ILCs), and predominantly mediated through cytokines. Antigen-dependent mucosal protection is provided by CD4^+^ T helper 17 (T_H_17), regulatory T (T_reg_), and CD8^+^ tissue-resident memory (T_RM_) and Tc17 cells. MAIT cells and other unconventional T cells, such as invariant natural killer T (iNKT) and gamma-delta T (γδT) cells, share features with both innate and adaptive cells. They can be activated in a TCR-dependent or independent manner (20, 48, 49, 55), and are important for preserving tissue integrity and function in homeostasis.

### Ontogeny and Tissue Residency

Human MAIT cells in blood have a tissue-homing effector-memory phenotype (3), but the ontogeny of cells in barrier surfaces is less clear. Murine tissue MAIT cells and iNKT are resident populations in homeostasis and following infection, expressing the transcription factor retinoic acid-related orphan receptor (ROR) γt (RORγt). After parabiosis, almost all thymic, and the majority of splenic, hepatic, lymph node and RORγt^+^ lung MAIT cells are host-derived, with some recirculation of RORγt^−^ lung MAIT cells (47, 56). Following *Staphylococcus epidermidis* (*S. epidermidis*) challenge similar frequencies of murine skin MAIT, γδT, and iNKT cells are host-derived and thus tissue-resident (35).

In humans however, it is unclear to what extent tissue MAIT cells are permanently resident. Expression of αE-integrin (CD103) associated with CD8^+^ T_RM_ cells is rare in blood MAIT cells (<3%) and common, but not universal, among MAIT cells within the oral and gastric mucosa (13, 15), skin (31, 57), and lungs (27–29) (**Table 1**). Thus tissues in health may represent a mixture of resident and recirculating MAIT cells, which could vary micro-anatomically: although most epidermal MAIT cells express CD103 and CLA (cutaneous lymphocyte antigen), only half of dermal MAIT cells express CD103 (31). Resident populations may behave differentially in disease – for example, among bronchoalveolar CD8^+^CD161^++^Vα7.2^+^ cells, which are mostly MAIT cells, only the CD103+ fraction is depleted in HIV infection (28).

Studies on the formation and longevity of tissue MAIT cells in humans are challenging. Remarkably, some MAIT cells do migrate into fetal small intestine, liver and lung as early as the 2^nd^ trimester - before exposure to the conventional commensal flora (25). Fetal tissue MAIT cells, unlike circulating MAIT cells in adults, are cycling in the steady state with appreciable Ki67 expression (25). It is unclear if these early tissue resident MAIT cells could persist into adult life, similar to other fetal tissue-resident cells (58). One study using HLA allele mismatching to discriminate between donor- and recipient-derived T cells following small bowel transplantation showed that although the majority of tissue MAIT (CD161^+^Vα7.2^+^) cells are derived from the host long term, donor-derived cells can be found over a year after transplantation (59); this suggests that adult mucosal MAIT cells can persist for prolonged periods. The dynamics may of course vary in tissues with different cellular turnover, and analogous to the differential proliferative capacity and function of resident and monocyte-derived macrophages in tissues (60), the function of long-lived and newly formed MAIT cells may be distinct.

It is also unclear if MAIT cells can leave tissues. Thoracic duct lymph-derived and blood MAIT cells are CCR7^−^ and present at comparable frequencies with overlapping TCR clonotypes (61), which suggests that MAIT cells in lymph directly derive from blood. This could imply either migration after transit through tissues, or direct CCR7-independent migration from blood through high endothelial venules. Further work is needed to understand the drivers for tissue migration, residency and persistence, including potential functional differences between long lived and nascent tissue MAIT cells.

### Tissue Phenotype and Cytokine Production

MAIT cell expression of the NK-cell marker CD161 and the transcription factor RORγt (*RORC*) are features shared with other barrier tissue-resident cells, including T_H_17, peripherally derived T_reg_ (pT_reg_), ILC3, iNKT and γδT cells (62–67). CD161^+^ T cells share a transcriptional program for innate-like cytokine-responsiveness in the absence of TCR triggering, through high expression of cytokine receptors (e.g., IL-12R, IL-18R) driven by the transcription factor promyelocytic leukemia zinc finger protein (PLZF) (45, 55). RORγt is crucial for barrier protective IL-17 production common to many mucosal lymphocytes (43, 68). Unusually, in homeostasis many human MAIT cells co-express the type-1 transcription factor T-bet (*TBX21*) and RORγt, whereas naïve SPF mice have two almost mutually exclusive populations (69, 70): nearly all murine tissue resident MAIT cells are RORγt^+^T-bet^−^ IL-17A producing MAIT17, with less numerous T-bet^+^RORγt^−^ IFN-γ producing MAIT1 found in the circulation (47). Following infection with intranasal Salmonella or Legionella, an increase in RORγt^+^T-bet^+^ lung MAIT cells is observed, suggesting that the diverse stimuli that human MAIT cells receive may explain part of the differences between species (71).

Functionally, human tissue MAIT cells are usually more activated than their circulating counterparts in homeostasis and capable of increased cytokine production (19, 23, 72) (**Table 1**). Additionally, barrier MAIT cells in the female genital tract and oral mucosa are predominantly CD8^+^ cells biased towards type-17 function (13, 32). Another study found that CD4^−^CD8^−^ MAIT cells in healthy endometrium have a more mature phenotype with higher RORγt and lower T-bet expression (33). This skewed tissue biased phenotype is present from early development as fetal small intestine MAIT cells produce more cytokines (IFNγ, IL-22) than their circulating counterparts in response to *Escherichia coli* (*E. coli*) (25). It is unclear however if this phenotype varies between barrier tissues in adults. Barrier specific heterogeneity is observed in mice: murine skin MAIT cells display a transcriptional profile distinct from lung or liver (35), and both colonic and lung MAIT cells produce higher IL-17 than non-barrier tissues, such as the liver and spleen (73). Ultimately, as IL-17A and IL-22 promote barrier integrity, it will be important to understand the relative contributions of pre-programmed transcriptional and environmental cues to MAIT cell heterogeneity observed in human tissues.

### Tissue Homeostasis and Tissue Repair

Given their anatomical location and similarity to other tissue-resident lymphocytes, it is perhaps unsurprising that MAIT cell effector function is important in tissue-homeostasis. This parallels tissue homeostatic roles for H2-M3 restricted CD8^+^ T cells (and T_regs_) in mouse skin (74, 75), and γδT cell populations in the lung and gut (76–78). In NOD mice, *Mr1*-deficient animals have impaired intestinal barrier integrity, implicating MAIT cells in maintaining surface homeostasis (79). This concept was further supported in a model of colonic graft versus host disease (GvHD); *Mr1*-deficient animals had increased proinflammatory donor-derived CD4^+^ T cell expansion and reduced tight junction expression (73). GvHD in human bone marrow transplant recipients is also associated with lower MAIT cell frequency (80, 81), again potentially implicating a role in maintaining mucosal health.

Further recent data from both mouse and human studies has identified an important role for MAIT cells in tissue repair. Constantinides et al. found that murine skin resident MAIT cells are enriched for a distinct tissue repair transcriptional signature, similar to that observed in the previously described H2-M3 restricted CD8^+^ T cells responsive to *S. epidermidis*-derived N-formylated peptides (35). To assess MAIT cell specific tissue repair *in vivo* without confounding skin γδT and H2-M3 restricted CD8^+^ cells which can perform analogous functions, *Tcrd* ^−/−^ mice were infected with a strain of *S. epidermidis* that fails to induce CD8^+^ H2-M3-recognizing T cells. Tissue repair in response to *S. epidermidis*, measured by epidermal tongue length growth after skin punch biopsy, was higher in *Tcrd* ^−/−^ compared to *Mr1* ^−/−^ *Tcrd* ^−/−^ mice, implicating the additional MAIT cell deficiency.

How do these data relate to human MAIT cells? Recently, murine and human MAIT cells were also found to have a shared tissue repair transcriptional profile (as seen with the H2-M3 restricted CD8^+^ T cells) on resolution of infection from *Legionella longbeachae* and with re-infection, suggesting significant functional parallels (48). Two additional studies in human MAIT cells showed activation of such tissue repair gene expression patterns predominantly following TCR-mediated triggering (20, 49). *In vitro* assays of wound healing also revealed a functional repair role for MAIT cell derived soluble factors, which could be blocked using anti-MR1 antibodies (20). Taken together all these studies suggest that a local repair program analogous to other tissue resident cell types is active in MAIT cells and likely triggered *in vitro* through encounter with microbiota. This is supported by the remarkable observation *in vivo* that direct topical application of the MAIT cell TCR ligand 5-OP-RU alone prior to skin injury, in the absence of additional cytokines, is sufficient to selectively induce cutaneous MAIT cell expansion and expedite tissue repair (35). Much more work is needed to define the importance of this in human disease and also the exact mechanisms through which this large panel of soluble mediators exert their impact.

In addition to tissue-repair functions in response to commensals in the absence of inflammation, there is evidence that innate-like T cells can regulate barrier surface homeostasis by shaping the microbial landscape. CD1d and intestinal iNKT cells influence murine intestinal homeostasis and microbial colonization, with reduced *Bacteriodales* colonization in iNKT-deficient mice (82). *Mr1*-deficient animals, which lack MAIT cells, have reduced intestinal microbial diversity, similar to that found in IL-17A deficient animals (73). Conversely, in obese mice MAIT cells seem to promote microbial dysbiosis and ileal barrier dysfunction. Fecal transplantation from obese *Mr1*-deficient animals reduces barrier permeability in mice fed a high-fat diet, and the microbiome of obese Vα19^+/−^ mice with a high MAIT cell frequency has lower *Bifidobacteriaceae* and *Lactobacillaceae* species (83). Given the importance of a diverse microbiome to human health, further work is needed on the interactions of MAIT cells and a healthy microbiome in maintaining tissue homeostasis.

These expanding tissue-specific functions raise the tantalizing possibility that similar to other innate-like lymphocytes, MAIT cell barrier functions may be more diverse than initially appreciated (84, 85). We know that γδT cells can remarkably promote stem-cell remodelling (86), adaptive thermoregulation in response to cold stress (87), and sympathetic nervous innervation (88). Furthermore, cytokine activated ILC3 can promote antigen specific CD4^+^ T cells responses directly in vitro through cell surface MHCII and co-stimulatory molecule expression (89). Indeed MAIT cells have the capacity to indirectly manipulate tissue adaptive responses, through dendritic cell maturation (90), and could potentially act as a sink for IL-7 similar to IL-7R^+^ ILC to limit homeostatic proliferation and preserve TCR diversity in neighboring tissue T cells (91).

In summary, MAIT cells in tissues are distinct from the population most frequently studied thus far in blood. The array of effector functions is expanding beyond the traditional cytotoxicity and cytokine production first described, and further understanding of the context in which these effector programs are engaged together with knowledge of how to modulate them are key to enable translation of MAIT cell biology to effective human therapeutics.

## Barrier Adaptation

### Metabolism

T cell development, differentiation, activation, function and survival are regulated by the cell intrinsic metabolism of glucose, amino acids and lipids (92–94). Nutrient availability differs between T cell compartments: circulating lymphocytes function in secondary lymphoid organs with high glucose and amino acid concentrations, whereas barrier tissues are more restrictive anatomically with variable nutrient composition. Permanent tissue-resident cells must therefore adapt to these niches for continued survival and proliferation which are also required for their tissue-specific effector functions.

Activated peripheral blood MAIT cells upregulate glucose uptake as glycolysis is required for their cytotoxicity and IFN-γ production (95, 96), and human circulating MAIT cells have reduced mitochondrial activity compared to CD161^−^CD8^+^ T cells (95). Amino acid metabolism is also crucial - peripheral MAIT cells express high levels of the L-type amino acid transporter, SLC7A5, and L-amino acid oxidase (96, 97). The metabolism of tissue MAIT cells has however not been explored.

Tissue MAIT cells may rely on OXPHOS, which correlates with TNF and IL-17 production in their circulating counterparts (96, 98). Given low tissue glucose concentrations, many resident immune cells are adapted to oxidative phosphorylation of fatty acids abundant in the skin and intestine (99). Tissue-resident but not circulating memory CD8^+^ T cells require these exogenous fatty acids for their survival, and upregulate transporters and fatty acid binding proteins (FABP) necessary for long term maintenance (100, 101). FABP isoform expression is tissue-specific, shared among resident T_RM_, IEL, ILC and γδT cells, with T_RM_ able to modulate isotype specific expression on relocation to a new environment (102); *Fabp4* and *Fabp5* expression are enriched among skin resident cells, whereas *Fabp1* is enriched among liver resident cells, including invariant NKT cells. Endogenous fatty acid metabolism is also important in IL-17 producing T_H_17 (103), with murine Tc17 cells and human IL-17 producing bronchoalveolar MAIT cells also enriched for genes in fatty acid and lipid metabolism (29, 104). It would therefore be logical to explore tissue MAIT cell mitochondrial and lipid metabolism in understanding their barrier specific effector functions.

### Tissue Stress

Tissue stress includes homeostatic perturbations in metabolic or environmental factors. Examples include insufficient nutrients or accumulations of potentially toxic byproducts, including oxidative stress from excessive free radicals. Barrier tissues with an active immune response frequently have minor homeostatic perturbations tolerated by resident immune cells.

Autophagy is a metabolic stress response that recycles intracellular proteins and provides an additional nutrient source advantageous in tissues and activating environments (93, 105). MAIT cells in the liver have higher basal autophagy compared to their circulating counterparts, which may be required given the higher mitochondrial depolarization observed in stressed tissue-resident cells (106). *In vitro*, inhibition of autophagy reduces acquisition of a tissue-resident phenotype in circulating CD8^+^ T cells (106), thus enhanced autophagy may be a requirement for MAIT cell tissue survival.

Tissue oxidative stress can also be mitigated in barrier tissues by xenobiotic transporters, such as multidrug resistance protein 1 (MDR1). MDR1 (ABCB1) is an ATP-binding cassette B1 drug resistance transporter expressed on IL-17 producing CD4^+^ T cells in the ileum, which protects against bile acid induced oxidative stress to maintain intestinal homeostasis (107); a subset of patients with ileal Crohn’s have loss of function in MDR1, highlighting an important role in controlling tissue inflammation. MAIT cells and other CD161^+^ T cells also express high levels of MDR1 (3, 45, 108), and it would be interesting to evaluate in future studies whether this has similar implications for their survival and function in toxin rich niches.

Tissue oxidative stress also produces free radicals and hydrogen peroxide (H_2_O_2_). H_2_O_2_ can be transported by the plasma membrane water channel, aquaporin 3 (*AQP3*), which is part of a core transcriptional signature shared among type-17 secreting NKT17, ILC3, γδT, and T_H_17 cells in mice (109). *Aqp3-*deficient T cells have impaired chemokine mediated trafficking to the skin (110), and *AQP3* expression is higher in human CD161^+^Vα7.2^+^ MAIT cells compared to circulating CD161^−^ cells expressing the same TCRα (46). It is therefore possible that tissue stress regulates MAIT cell migration and survival in inflammatory tissues such as the skin.

Finally, given the often overlapping functions, some adaptations may serve to prune the tissue response to only the most appropriate cells. Murine T_H_17, iNKT and T_RM_ are enriched for the purinergic receptor, P2RX7, which recognizes extracellular purines (ATP, NAD^+^) released after cell lysis and has cell type specific effects (111–113). Purines released from high microbial turnover can regulate barrier specific immune cell function, promoting murine T_H_17 differentiation (111) but inhibiting human ILC3 IL-22 production (114). P2RX7 expression on tissue iNKT and T_RM_ however induces pyroptosis and limits immunopathology from bystander activation (113, 115). As purinergic receptor expression is downregulated on TCR engagement, release of purines from tissue damage preferentially depletes bystander activated T_RM_ (115). Over time this could serve to shape the barrier immune response by conserving predominantly T cells specific to regularly encountered antigens. Given their presence in tissues and potential for bystander activation in response to cytokines, MAIT cell function may also be shaped by variable adaptations to tissue damage. Indeed, one could imagine that as a mammal ages, MAIT cell TCR-dependent responses could be superseded by antigen-specific T_RM_ populations.

## Barrier Sensing

### Microbiome

Barrier surveillance of the commensal microbiome is essential for tissue immunity in homeostasis through shaping the composition and phenotype of innate and adaptive cells (116–118). Numerous mucosal cell types are perturbed in germ-free (GF) and antibiotic treated mice, including RORγt-expressing tissue T_reg_, T_H_17 and innate-like IL-17 producing γδT cells (119). Conversely cohousing laboratory mice with wild mice to induce a more diverse microbiome promotes a human adult-like immune composition, with increased tissue innate and differentiated memory CD8^+^ T cell populations (120).

We have known MAIT cells are also reliant on the microbiome since Treiner *et al.* discovered that they were absent in the lamina propria of GF mice (2). It was subsequently found that metabolites from riboflavin-synthesizing commensals, which engage the MAIT cell TCR, are necessary for most stages of MAIT cell intra-thymic development and subsequent peripheral expansion (56, 121). Accordingly, the dominant murine population of RORγt^+^ MAIT17 dependent on TCR-triggering for proliferation and function are depleted in the thymus and tissues of GF-mice, skewing the response to IFN-γ production (56). Recolonization of GF mice with microbes can rapidly restore this RORγt^+^ MAIT population. Remarkably, metabolites from skin riboflavin-synthesizing commensals, even in the absence of bacteria, can drive intra-thymic MAIT cell development remotely (56), in addition to sustaining the development and function of local skin-resident MAIT cells (35). There is however a narrow neonatal window until 3 weeks of age where recolonization of GF mice can restore MAIT cell development; recolonization of adult mice with bacteria after 7 weeks does not increase MAIT cell frequencies in the skin. Although they have complementary functions, this may be due to a finite niche for competing innate-like T cells shaped by the early microbiome; *Tcrd*-deficient animals have increased tissue-resident iNKT and MAIT cell populations (35), *Cd1d*-deficient animals have increased splenic and thymic MAIT cells (121), while GF mice have increased iNKT cells (122). A competing or compensatory interaction is also supported by the massive expansion of Vδ2^+^ T cells in a patient with a homozygous point mutation in MR1 and MAIT cell deficiency (123). Supporting complementary functions, human blood MAIT cell and iNKT cell frequencies positively correlate (124, 125). Additional work is needed to clarify the relationship between innate-like T cells, and whether this is influenced by age, disease and ligand abundance.

In humans, with a much lower frequency of tissue iNKT and γδT cells, it remains to be seen if this window period for reconstitution and niche exist in adulthood. BAL MAIT cells depleted in HIV are increased with ART; as ART partially restores a dysregulated lung microbiome (126), it is tempting to speculate this contributes to MAIT cell reconstitution. Peripheral MAIT cells however are not reconstituted by ART (21, 24). Long term reconstitution is also seen after allogeneic hematopoietic stem cell transplantation (81), dependent on the microbiome and potentially continued thymic output given the negative correlation with age.

#### Microbial Diversity and Pathogenicity

The mammalian microbiome is diverse and heterogeneous, varying between sites with different microenvironments. Skin hair follicles, sweat glands and sebum promote distinct commensals and immune responses compared to the intestine (52, 127, 128); diet, age and antimicrobials all shape the gut microbial landscape (129). Furthermore, dysbiosis can result from changes in the tissue microenvironment which drives the expansion of more suitably adapted commensals.

The appreciation of different microbial phyla to MAIT cell expansion and function is expanding. A screen of bacterial species *in vitro* found that the capacity to stimulate MAIT cells correlated with riboflavin secretion (130). Colonization of GF mice with *Proteus mirabilis* alone in the neonatal period is sufficient for MAIT cell expansion in the skin and lungs (35). In reality we have communities of microbes and there is evidence that increased microbial diversity is associated with improved MAIT cell reconstitution after allogeneic hematopoietic stem cell transplantation (81). This could partly be through a reduction in their activation induced cell death as in *vitro* microbial diversity has been shown to reduce MAIT cell activation (131). Testing common intestinal commensals *in vitro* has demonstrated that MAIT cell activation correlates with net riboflavin secretion, with higher diversity resulting in predominant riboflavin uptake and thus lower presentation to MAIT cells (131). This is supported by observations in apical periodontitis oral mucosa, where prominent riboflavin-expressing taxa correlate negatively with Vα7.2-Jα33 and IL17A transcripts (132). Furthermore, *Il17a-*deficient mice, which have microbial dysbiosis and reduced barrier protection, actually have increased MAIT cell frequencies in the lung and colon. As *IL17a*-deficient mice have increased *Candidatus Homeothermaceae* and *Bacteriodaceae*, it is tempting to speculate that the composition of the microbiome is crucial for MAIT cell expansion (73). As microbial diversity varies between tissues in health and disease, normally high in healthy colon and reduced in dysbiosis and metabolic diseases, this could be a mechanism to manipulate MAIT cell function and through which they may contribute to pathology.

In addition to diversity, healthy tissues are characterized by an intact barrier, disruption of which induces inflammation and could impact MAIT cell function. Most microbes are commensals living in symbiosis with the host; pathogens induce barrier disruption, inflammation and cytokine production. For example, colonization with commensal *S. epidermidis* does not induce inflammation and is important for tissue homeostasis and a MAIT cell tissue repair signature (35). However, stimulation of human MAIT cells *in vitro* with cytokines in addition to TCR engagement promotes an antimicrobial program, including the cytokine IL-26, capable of directly killing extracellular bacteria (133), and effector recruiting chemokines CXCL9 and CXCL10 (20, 49). Thus, the pathogenicity of the human microbiome could tune the MAIT cell response, and further studies should assess the various pathogen-specific factors which induce antimicrobial rather than more tolerant repair effector programs.

#### Microbial Metabolism and Environment

Microbial metabolism is also shaped by the tissue microenvironment and could tune MAIT cell activation. One mechanism is through availability of TCR ligands derived from riboflavin synthesis: heat stress in *Streptococcus pneumoniae* induces expression of the riboflavin operon (134); and acid stress increases purine and folate metabolism, as well riboflavin uptake (19, 131). Bacterial co-culture conditions influence their capacity to activate human MAIT cells *in vitro* (19); hypoxia, simulating the low oxygen tension in colonic crypts, stationary growth phase and hypercarbia increase MAIT cell activation, whereas hypoglycemia, acetate and pyruvate inhibit bacterial control (19). Even chemicals and pesticides, which cause gut dysbiosis on ingestion of food, have been found to increase *E. coli*-induced MAIT activation (135). MAIT cells could therefore survey the nature and state of the microbiome as a proxy measure of tissue health.

Non-riboflavin microbial metabolites could also modulate tissue MAIT cells, contributing to tissue homeostasis or pathogenic inflammation. *Lactobacilli*, enriched in the female genital tract, produce high levels of L(+)-lactate; and both lactate and *Lactobacilli*-derived factors dampen *Staphylococcus aureus* (*S. aureus*)-induced MAIT cell activation in whole PBMC (136). Given that the antibacterial response of MAIT cells resident in the female genital tract is biased towards IL-17 and IL-22 production compared to their circulating counterparts (32), it is tempting to speculate that microbial derived factors at barrier surfaces might directly skew MAIT cell responses.

There is evidence that other products of bacterial metabolism, short chain fatty acids (SCFA), can directly modulate barrier RORγt^+^ immune cell responses (137). Acetate, propionate and butyrate are products of dietary fiber fermentation which signal via G-protein coupled receptors (GPCR) to inhibit histone deacetylases (HDACs) (137). These SCFA directly promote barrier preservation and can reverse some of the immune dysregulation in GF-mice; SCFA rescue the colonic T_reg_ depletion seen in GF mice (138) and are capable of augmenting RORγt^+^T_reg_ expansion (139–141) and ILC3 IL-22 production (142). Barrier cells such as ILC3 can directly sense acetate through *Ffar2 (*free fatty acid receptor 2) (142), which interestingly is also enriched transcriptionally in murine skin MAIT cells compared to CD4^+^ T cells (35). As SCFA reduce MAIT cell antimicrobial function *in vitro* (19), it would be of interest to determine if microbial metabolites could be sensed and tune MAIT cell responses in a TCR-independent manner.

### Dietary Factors

In addition to the microbiome, mucosal immune cells are capable of directly sensing chemicals, including nutrients in the mammalian diet (39). Dietary-derived metabolites, particularly lipophilic compounds, rapidly diffuse and bind to intracellular ligand-dependent transcription factors and can regulate tissue resident cells (143); these include receptors for vitamin A (retinoic acid receptor, RAR), vitamin D (vitamin D receptor, VDR), and tryptophan metabolites (aryl-hydrocarbon receptor, AhR). Given their often-shared function and location, MAIT cells may also be regulated by these dietary factors.

#### Vitamin A

The fat soluble Vitamin A is enriched in human intestine and is important for mucosal health (39). Dietary vitamin A, as all-trans-retinol, retinyl esters, or β-carotene, is metabolized to bioactive *all-trans-*retinoic acid (ATRA) and *9-cis-*retinoic acid (144), which bind to nuclear retinoic acid receptors RARα (*RARA*), RARβ (*RARB*), and RARγ (*RARG*) (39). In mice RA maintains barrier homeostasis by directly modulating T_reg_, T_H_17 and ILC3 function. RORγt^+^pT_reg_ development, homing and differentiation are RA dependent (145–148), with dietary deficiency promoting T_H_17 mediated tissue pathology (141). Similarly ILC3 gut homing (149), plasticity (150), and IL-22 mediated protection in DSS colitis are RA dependent (151). In humans, ATRA directly increases CD161 and gut homing CCR9 expression in a population of RORC^+^CD161^+^ colonic T_reg_ associated with tissue repair (64). Vitamin A deficiency is also strongly associated epidemiologically with severe mucosal infections, which may partly be through direct effects on barrier protective immune cells.

The role of RA in innate-like T cells is less clear. RA reduces invariant NKT induced-sterile tissue damage by inducing P2RX7 expression, rendering bystander but not TCR-activated cells more susceptible to extracellular ATP-induced pyroptosis (152). γδT cell function is also reduced: CD27^−^γδT cell IL-17 production is inhibited by RA through reduction in IL-1R, IL-23R, and pSTAT3 expression (153). In IBD tissue however, RA levels correlate with increased γδT and MAIT cell function (IL-17, IFN-γ) (154). As *RARG* is upregulated in blood MAIT cells relative to conventional T cells (46), vitamin A could conceivably modulate intestinal MAIT cell migration and function to ultimately maintain mucosal integrity in an analogous manner to neighboring CD161^+^ T cells.

#### Vitamin D and Cholesterol Metabolites

The lipophilic oxysterol derivate **Vitamin D** can be derived from the diet or photochemically synthesized in the skin (39), and binds to its heterodimeric receptor, composed of VDR and the retinoid X receptor (RXR). Immune cells, particularly those in the intestine and skin, are enriched for expression of the nuclear VDR which is reduced in inflammatory bowel disease and implicated in the moderation of mucosal inflammation (39, 155). VDR expression is upregulated on TCR signaling and decreases type 17 associated immunopathology in humans and mice, increasing the ratio of T_reg_: T_H_17 (156, 157), inhibiting ILC3 IL-23R expression (158), and directly competing with NFATc1 for binding to the *IL17A* promoter (159). *Vdr* ^−/−^ mice also have impaired iNKT and CD8αα^+^ IEL development, suggesting a broad role in tissue immunity (159–161).

MAIT cell frequency and function may also be subject to regulation by vitamin D. MAIT cells triggered through their TCR upregulate *VDR*, either *in vitro* in humans or during acute *Legionella longbeachae* infection in mice (48). In asthmatic patients, seasonal fluctuations in peripheral MAIT cell frequency correlate with serum phytochemically derived vitamin D_3_ levels and peak in August (162). In cystic fibrosis patients however, although baseline serum vitamin D3 correlates with peripheral MAIT cell CD38 expression, there was a trend for reduced MAIT cell frequency in those receiving oral vitamin D supplementation (163). Dietary and photochemically-derived vitamin D may differentially regulate MAIT cells in different compartments and activation states – whether responding to commensals in homeostasis or during active inflammation.

Other cholesterol derivatives of host or microbial metabolism, including **oxysterols**, are abundant in the intestine and can act as RORγt ligands to promote development and function of RORγt^+^ intestinal cells (164). Stromal cells produce 7-α,25-hydrocycholesterol, which binds to GPR183 expressing ILC3 to promote their migration in homeostasis (165). As tissue IFN-γ producing MAIT cells transcriptionally express the oxysterol receptor GPR183 (29), oxysterol sensing may also functionally regulate MAIT cells.

**Bile acids** are cholesterol-derived surfactants crucial for fat digestion that bathe the ileum as part of the enterohepatic circulation and regulate both the microbiota and mucosal immunity (166). Secondary bile acids derived from microbial metabolism, including deoxycholic acid (DCA) and lithocholic acid (LCA), can directly promote mucosal homeostasis by increasing colonic FOXP3^+^ RORγt^+^ T_reg_ (167). A screen of secondary bile acids also found that LCA derivatives can reduce the T_H_17:T_reg_ balance in the intestinal lamina propria, by directly blocking RORγt-induced T_H_17 differentiation and promoting T_reg_ *Foxp3* expression and differentiation in a mitochondrial ROS-dependent manner (168). MAIT cell activation and PLZF expression negatively correlate with serum concentrations of conjugated bile acids in teenage children, and *in vitro* bile acids inhibit MAIT cell activation in response to *E. coli* (169), so it would be important to explore whether intestinal bile acids promote homeostatic MAIT cell responses against commensals within a healthy functioning symbiotic intestinal environment.

#### Aryl Hydrocarbon Receptor

Aryl hydrocarbon receptor (AhR) is a conserved ligand activated transcription factor highly expressed by cell types at barrier surfaces, in keeping with its role as an environmental sensor promoting mucosal integrity. Physiological AhR ligands include: indole-derived ligands from dietary cruciferous vegetables; host and microbe-derived tryptophan-metabolites (e.g., kynurenine); and exogenous chemicals (40). Initially discovered and enriched in intestinal T_H_17 and T_reg_ (41, 170), AhR signaling is also important for the function of mucosal IL-17 producing γδT, iNKT and ILC3 (66, 171, 172). Sensing of diverse environmental signals promotes T_reg_ differentiation, IL-22 production, ILC3 survival and IEL homeostasis, thus promoting barrier integrity (173). *Ahr*-deficient mice have dysfunctional skin and intestinal γδT cells and absent IEL (172, 174), with reduced capacity for T_H_17 differentiation and IL-22 secretion. AhR expression in CD8^+^ T cells is crucial for T_RM_ and IEL persistence in tissues (175, 176), while cytokines upregulate AhR expression in NK cells and iNKT to promote cytotoxicity and IL-22 production respectively (66, 177).

The role of AhR in MAIT cells has only been tentatively explored. *Ahr* is dispensable for MAIT cell thymic development in mice (56). In HIV patients on ART, increased tryptophan catabolism and generation of the AhR ligand, kynurenine correlates with lower peripheral blood MAIT cell frequency and higher frequency of T_reg_ (178). As *AHR* expression is higher in bronchoalveolar MAIT cells compared to matched circulating cells in children with pneumonia (29), further work should explore specifically whether tissue MAIT cells are selectively regulated by AhR in a similar manner to other IL-22 producing cells in particular.

#### Lipids

The predominant calorie source of diet can influence barrier immunity in mice. A high glucose diet exacerbates colitis by increasing mitochondrial metabolism to drive T_H_17 differentiation (179). Mice fed a high fat diet also have increased T_H_17 differentiation through induction of the lipid sensitive kinase, acetyl co-A carboxylase 1, crucial for de novo FA synthesis and oxidative phosphorylation (103, 180). A ketogenic high fat diet however protects against influenza challenge and promotes improved lung barrier integrity associated with early lung γδT cell recruitment, expansion and barrier type-17 function (181). In addition to diet, tissue free fatty acids have also been shown to induce a regulatory phenotype in iNKT (182). Lung type-17 MAIT cells in the context of pneumonia are enriched in genes for OXPHOS, glycolysis, lipid efflux and translocation, while other MAIT cells are enriched in genes for steroid metabolism, fatty acid synthesis and lipid uptake (29). Further studies should investigate the regulation of MAIT cell function by lipids and metabolism.

### Tissue Environment

Tissue immune cellular and soluble mediators, particularly cytokines, manipulate the function of MAIT cells and other resident populations (4). This is further nuanced by the confined shared niche occupied by resident cells which compete for space and local survival signals (175). Tissue inflammation and infiltration of metabolically active, cytotoxic cells into this niche can disrupt homeostatic regulation by depleting nutrients (glucose, amino acids) and oxygen, producing waste products such as reaction oxygen species and lactate which contributes to tissue acidosis (183, 184). Resident immune cells are themselves in turn tuned by these non-immune tissue parameters.

#### Oxygen Sensing

Oxygen tension and regulation varies *in vivo*: blood and primary lymphoid organs have tightly regulated levels, whereas physiological hypoxia is observed in tissues such as the skin and intestine (38, 52, 185). Microbes can also indirectly induce colonic oxygen consumption through SCFA (186). Hypoxia regulates immune cells directly by preventing cytosolic degradation of the oxygen sensing transcription factor, hypoxia-inducible factor (HIF1A). T cell upregulation of *HIF1A* expression is STAT3-dependent and promotes CD8^+^ T cell effector functions (187), T_reg_ plasticity (188, 189), and T_H_17 differentiation through induction of glycolysis, *Rorγt* expression and *Foxp3* proteasomal degradation (190, 191).

*In vitro* sorted human MAIT cells co-cultured with proximal tubular epithelial cells are more activated in hypoxic conditions, with increased cytotoxicity albeit no difference in cytokine production (192). In children with pneumonia, bronchoalveolar MAIT cells have higher *HIF1A* expression compared to their blood counterparts which correlates with their capacity for increased IL-17 production (29). Furthermore, the tissue repair signature enriched in MAIT cells engaged through their TCR includes upregulation of *HIF1A* in addition to factors associated with angiogenesis (*VEGFA, PDGF2, CSF2*) (48). It would seem plausible that tissue MAIT cells, likely to experience local hypoxia during the course of an immune response, could tune their effector functions accordingly to ultimately induce tissue repair and improve oxygenation.

#### pH

Although circulating pH is homeostatically maintained around pH 7.4, deviations are seen in tissues: healthy skin is acidic due to a high free-fatty acid content; and inflammation drives tissue acidosis through glycolytic products (52). Many immune cells possess mechanisms for proton sensing, including acid-sensing ion channels (ASIC), transient receptor potential (TRP) channels, and GPCRs (193). Among T cells, human MAIT cells and other CD161^+^ T cells share functionality and a conserved transcriptional signature by bulk microarray, which includes enrichment for two candidate GPCR proton sensors, *GPR65* and *GPR68* (45, 194, 195). GPR65 may play an important role in *RORγt^+^* T cells; the *Gpr65* promoter has a *RORγt* binding site (196), and Gpr65 expressing T cells regulate the development of EAE in mice which is driven by type 17 inflammation (197). Naïve *Gpr65* ^−/−^ CD4^+^ T cells differentiated under T_H_17 conditions, or memory *Gpr65* ^−/−^ CD4^+^ T cells reactivated with IL-23 produce less IL-17A *in vitro*, and the adoptive transfer of *Gpr65* ^−/−^ **CD4^+^ T cells into *Rag1* ^−/−^ recipients prior to EAE induction markedly delays and reduces disease (197). Another study however found that *Gpr65*-deficient mice develop exacerbated EAE, which was lost in the absence of iNKT (198); functionally deficient *Gpr65^gfp/gfp^* but not *Cd1d^–/–^ Gpr65^gfp/gfp^* mice develop more severe disease compared to wild-type. It is particularly interesting to note that murine *Gpr65* expression is important for CD4^+^ T survival in culture and highest in iNKT, followed by γδT and NK cells, suggesting a homeostatic role for acid sensing in innate-lymphoid cells. MAIT cells were not assessed in this study, but in humans share similarities with and are a hundred times more common than iNKT (67), thus may represent the most prominent GPR65 expressing cell. Indeed type 17 bronchoalveolar MAIT cells in children with pneumonia are enriched for *GPR65* expression so future studies should explore whether acid-sensing can promote MAIT cell mucosal function (29).

#### Lactate

Increased lactate is concomitantly seen with acidosis in inflammation, and can be directly sensed by CD4^+^ T and CD8^+^ T cells through SLC5A12 and SLC16A1 transporters respectively; these function to inhibit T cell migration and potentially promote tissue retention (199–201). However in mice, cells with low glycolytic capability, including murine iNKT, show reduced survival under high lactate conditions *in vitro* (202). MAIT cells and other IL-23R^+^ lymphocytes could also be indirectly regulated by lactic acid augmentation of TLR-induced IL-23p19 production (203), which would skew towards type 17 responses in tissues (32, 204).

#### Temperature

Fever is a conserved response to infection and autoinflammatory disease across species. Although core temperature is rigorously regulated, peripheral tissues such as the skin where MAIT cells reside, are prone to deviations (205). High temperatures have long been known to enhance human lymphocyte proliferation and cytotoxicity *in vitro* (206), as well as CD8^+^ T cell differentiation and CD4^+^ T cell activation through increased membrane fluidity and reduced co-stimulation thresholds (207, 208). In mice, antipyretics (aspirin, ibuprofen) inhibit T_H_17 differentiation, with high temperatures selectively promoting inflammatory T_H_17 differentiation and increased lung neutrophil recruitment (209). Pulmonary MAIT cells and IL-17A producing innate-like T cells clearly protect against bacterial and viral infections in mice, which become pyrexial during the course of a normal immune response (210–212). It is unclear if pharmacological or pathological alteration of this normal febrile response, or significant exposure to cold environments, could modulate the response of tissue MAIT cells.

#### Electrolytes and Osmotic Stress

Similar to pH, electrolytes such as sodium, potassium, and chloride are normally tightly regulated in blood. Elevated extracellular potassium is, however, found in necrotic tissues and tumors, which paralyses human cytotoxic T cell responses (213). It is also appreciated that salt (NaCl) concentration can be enriched in barrier tissues such as the skin, particularly during inflammation (44, 52, 214). High salt diet increases EAE severity in mice due to increased T_H_17 differentiation from naïve precursors; direct salt-sensing ultimately induces T_H_17 IL-23R expression (215, 216) and inhibits T_reg_ differentiation (217, 218).

In humans, high salt *in vitro* augments both naïve CD4^+^ T_H_17 polarization and memory CD8^+^ T cell IL-17A production in response to TCR-activation in the absence of polarizing cytokines (219). Interestingly, the skin of patients with atopic dermatitis has higher salt concentrations, and salt promotes both T_H_2 and T_H_17 cytokine production and skin-homing CCR8 expression by TCR-activated memory CD4^+^ T cells, thus potentially linking the environment with pathogenic mucosal T cell responses (219). Salt also indirectly regulates mucosal T cell function through differential production of polarizing cytokines: osmotic stress increases macrophage IL-1β production and Th17 generation in mice (220); and humans with a fixed high salt diet have increased plasma IL-23 (221). As MAIT cells are IL-23^+^ T cells in the skin, it would be interesting to determine whether their responses are also skewed in a similar way by the tissue electrolyte composition and if this contributes to disease.

#### Neuroendocrine System

The dense peripheral neuronal network underlying barrier surfaces co-ordinate rapid often reflex responses to external insults, such as itch, pain or cough reflex. Remarkably peripheral nerves directly regulate tissue immunity through soluble factors, including neuropeptides: neuromedin U (NMU) modulates ILC2-mediated tissue protection (222, 223); vasoactive intestinal peptide (VIP) increases ILC3-mediated epithelial barrier protection (224, 225); and catecholamines have inhibitory and stimulatory effects on ILC2 and NK cells respectively via adrenoceptor beta-2 (ADRB2) (226). The enteric nervous system can also indirectly control resident lymphocytes, through nociceptor induced IL-18 and IL-23 expression (227, 228). Given their location within this neuroimmune network, and expression of relevant receptors for cytokines and neuropeptides transcriptionally, MAIT cells could be subject to rapid manipulation by the nervous system.

Growth factors also regulate tissues and could influence MAIT cells. One example is insulin-like growth factor 1 (IGF-1) signaling, which promotes STAT3 signaling and aerobic glycolysis to increase type-17 effector functionality of T_H_17 and ILC3 (229). IGFbp4, an important modulator of IGF1 signaling, is enriched in murine RORγt^+^ T_H_17, T_reg_, and ILC3. In humans, *IGFBP4* is enriched in CD161^+^ T cells, so may be an important regulator of MAIT cell type-17 functionality (45). Indeed insulin resistance and fasting insulin levels in obese children correlate with circulating IL-17A producing MAIT cells (230), which may imply that feedback circuits regulating tissue glucose metabolism play an important role in skewing MAIT cell function.

#### External Environment

**UV-light** can regulate the immune system (231), partly through photochemical synthesis of vitamin D. Additionally, UV light dampens inflammatory pathology in psoriasis and has been shown to degrade numerous photosensitive MAIT cell ligands, including folic-acid derived 6-FP (6-formyl-pterin) (232). Given the unstable nature of MAIT cell ligands, the impact of light on skin MAIT cell responses to commensals in particular deserves further attention.

Finally, the **circadian rhythm** has a role in entraining barrier RORγt^+^ cells (233). Clock genes regulate the *RORC* promoter to dictate T_H_17 differentiation (234) and ILC3 function (235–237); and disruption of the light-dark cycle in mice exacerbates T_H_17 IL-17A-dependent DSS colitis. Pathway enrichment for innate-like T cells suggests that circadian clock regulation is a shared feature among human innate-like T cells, with transcription factors *ARNTL* (encoding BMAL), *RORA*, *PER1*, and *CRY1* enriched among MAIT, iNKT, and Vδ2^+^ γδT cells (238). As circadian biology regulates mammalian behavior and exposure to environmental factors, including food, this could be particularly relevant to mucosal MAIT cell function.

## Conclusion

**Figure 2:**
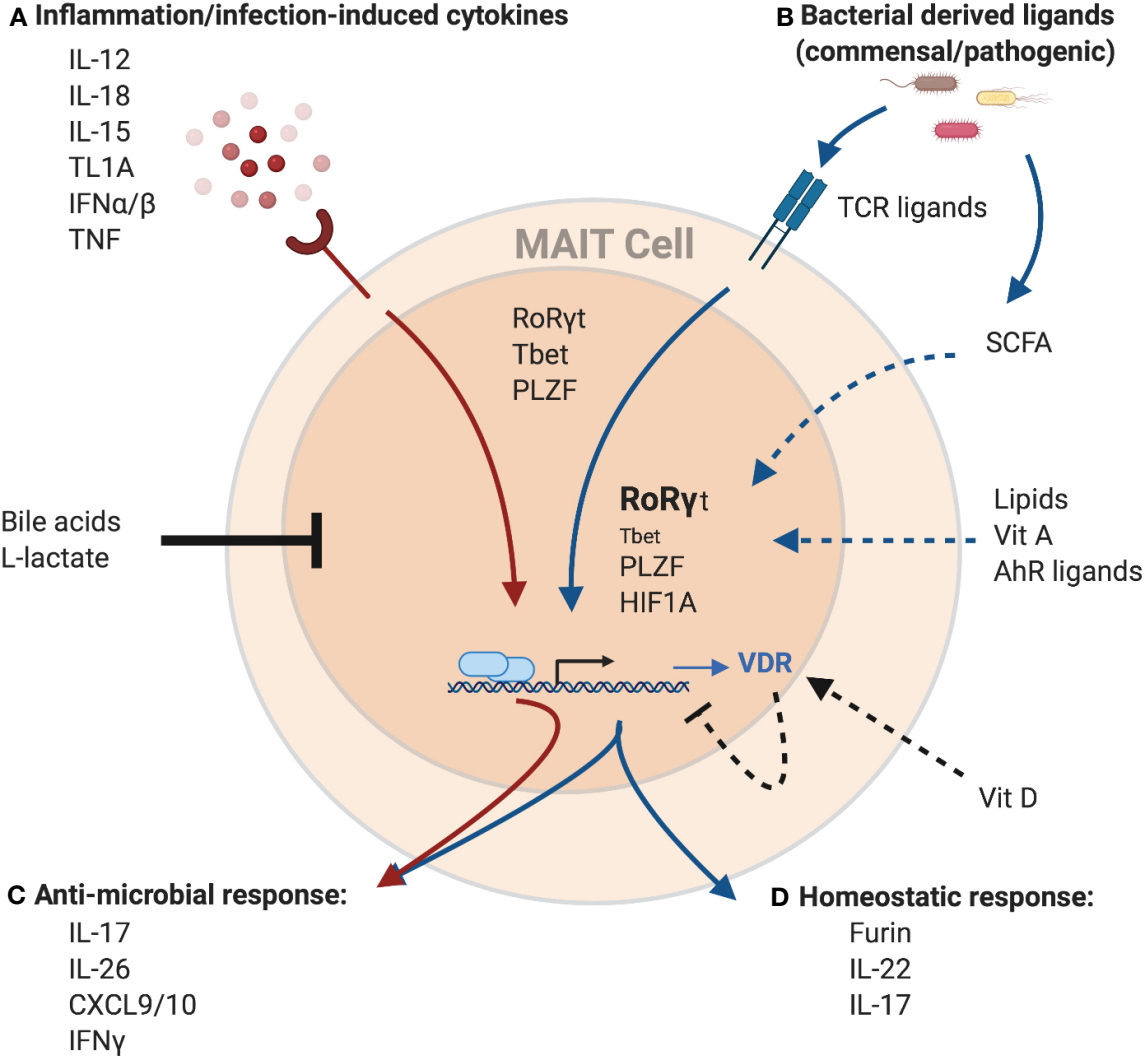
Potential regulation of the MAIT cell transcriptome and effector function by environmental cues. MAIT cells can be activated **(A)** independent of TCR-ligands by cytokines, or **(B)** through TCR-mediated recognition of microbial-derived riboflavin derivates presented by MR1. These signals can work both independently and synergistically to induce a spectrum of different effector programs. Cytokine-mediated MAIT activation results in the induction of a strong anti-microbial program **(C),** including the production of cytokines like IFNγ, IL-26 and members of the IL-17 family as well as pro-inflammatory chemokines like CXCL9 and CXCL10. These antimicrobial functions are further amplified with concurrent TCR signaling. TCR signals result in the induction of a homeostatic response **(D)**, including cytokines associated with barrier maintenance (IL-22, IL-17), and proteins associated with tissue repair, such as the endoprotease furin. MAIT cells effector functions are controlled by the transcription factors PLZF, RoRγt, and Tbet. Importantly, while PLZF expression within MAITs is stable, expression of the homeostatic effector program is associated with increased expression of RoRγt and decreased expression of Tbet. Finally, TCR-mediated MAIT cell activation also leads to expression of HIF1A, another transcription factor associated with tissue repair. In addition to TCR-ligands and cytokines, several other factors have the potential to modulate MAIT cell activation. Bile acids and L-lactate were shown to generally reduce MAIT cell responses, while binding of Vitamin D to its receptor (VDR), the expression of which is upregulated in MAIT cells in response to TCR-signaling, has the potential to specifically inhibit the homeostatic response. In contrast, recognition of several other metabolites including AhR ligands, Vitamin A and lipids was associated with the expression of homeostatic effector molecules in other T cell populations and hence, could positively influence the expression of these molecules in MAIT cells as well. Similarly, short-chain fatty acids (SCFA), a product of bacterial metabolism, were shown to stimulate production of IL-22 and expansion of RORγt-expression lymphocytes in other immune cells, while reducing the antimicrobial function of MAIT cells, which could overall present a mechanism to preserve tissue homeostasis. Created with Biorender.com.

MAIT cells serve an increasingly appreciated role in barrier tissues, yet the full range of effector functions remain to be determined. Similar to other mucosal lymphocytes, they are engaged in cross-talk with the tissues and microbiome via their TCR and through cytokine receptors (**Figure 2**). The context of this cross-talk in tissues, in addition to the array of increasingly recognized signals sensed by resident lymphocytes, suggest that other factors may influence MAIT cell activation, function, and plasticity. Indeed, these environmental factors were identified with mouse models that may have missed the impact on MAIT cell biology as these cells are infrequent in murine mucosal tissues. Humans, however, have an abundance of MAIT cells and in contrast to laboratory mice, are exposed to phenomenally diverse environmental factors unique to each individual. Exploring local environmental factors in addition to fixed pre-programmed factors in the investigation of MAIT cell tissue biology will be crucial to understanding the variability in humans and could pave the way for personalized therapies in the context of disease.

## Author Contributions

AA conceived the review, conducted the literature review, and wrote the bulk of the manuscript. DP contributed to writing of the manuscript. C-PH edited and revised the manuscript, and created the figures. PK contributed to the planning, editing, and scope of the review. All authors contributed to the article and approved the submitted version.

## Funding

AA is supported by a Wellcome Trust Clinical Research Training Fellowship [216417/Z/19/Z]. C-PH is supported by the Wellcome Trust [WT109965MA, awarded to PK] and the Deutsche Forschungsgemeinschaft (project number 403193363). PK is funded by the Wellcome Trust [WT109965MA]; the Medical Research Council (STOP-HCV); a National Institute of Health Research Senior Fellowship, the National Institute of Health Research Oxford Biomedical Research Centre, and the National Institutes of Health (U19 I082360).

## Conflict of Interest

The authors declare that the research was conducted in the absence of any commercial or financial relationships that could be construed as a potential conflict of interest.
